# Hif-1α Overexpression Improves Transplanted Bone Mesenchymal Stem Cells Survival in Rat MCAO Stroke Model

**DOI:** 10.3389/fnmol.2017.00080

**Published:** 2017-03-29

**Authors:** Bingke Lv, Feng Li, Jianbang Han, Jie Fang, Limin Xu, Chengmei Sun, Tian Hua, Zhongfei Zhang, Zhiming Feng, Xiaodan Jiang

**Affiliations:** Department of Neurosurgery, Zhujiang Hospital, Southern Medical University, The National Key Clinical Specialty, The Engineering Technology Research Center of Education Ministry of China, Guangdong Provincial Key Laboratory on Brain Function Repair and RegenerationGuangzhou, China

**Keywords:** Hif-1α, bone mesenchymal stem cells, cerebral infarction, autophagy, AMPK/mTOR signaling

## Abstract

Bone mesenchymal stem cells (BMSCs) death after transplantation is a serious obstacle impacting on the outcome of cell therapy for cerebral infarction. This study was aimed to investigate whether modification of BMSCs with hypoxia-inducible factor 1α (Hif-1α) could enhance the survival of the implanted BMSCs. BMSCs were isolated from Wistar rats, and were infected with Hif-1α-GFP lentiviral vector or Hif-1α siRNA. The modified BMSCs were exposed to oxygen-glucose deprivation (OGD) condition, cellular viability and apoptosis were then assessed. An inhibitor of AMPK (compound C) was used to detect whether AMPK and mTOR were implicated in the functions of Hif-1α on BMSCs survival. Besides, ultrastructure of BMSCs was observed and the expression of autophagy markers was measured. The modified BMSCs were transplanted into middle cerebral artery occlusion (MCAO) model of rats, and the cerebral infarction volume and neurological function was assessed. The results indicated that Hif-1α overexpression protected OGD induced injury by promoting cellular viability and inhibiting apoptosis. AMPK was activated while mTOR was inactivated by Hif-1α overexpression, and that might be through which Hif-1α functioned BMSCs survival. Hif-1α overexpression promoted autophagy; more important, compound C abolished the induction of Hif-1α on autophagy. Transplantation of the overexpressed Hif-1α of BMSCs into the MCAO rats reduced brain infarct volume and improved neurobehavioral outcome; besides, it inhibited pro-inflammatory cytokines generation while promoted neurotrophin secretion. In conclusion, Hif-1α might be contributed in the survival of BMSCs by regulating the activation of AMPK and mTOR, as well as by promoting autophagy.

## Introduction

Bone mesenchymal stem cells (BMSCs) are a population of multipotent cells, which can be easily harvested from autologous donors without ethical concerns and rapidly expanded *in vitro* (Pittenger et al., 1999). BMSCs transplantation has great potential for repairing and remodeling the neurovascular structure after ischemic brain injury (Tang et al., 2014), and the efficacy is positively correlated with the number of BMSCs grafted (Chen et al., 2001). Nevertheless, the survival rate of simple transplantation of BMSCs in ischemic tissues is unsatisfactory, and the poor cellular viability of the transplanted BMSCs largely limited the reparative capacity of those *in vivo* (Mangi et al., 2003). Thus, strategies to improve BMSCs survival is urgently needed which will be helpful for treating cerebral infarction.

Hypoxia-inducible factor 1 (Hif-1) is a master regulator of the cellular response to hypoxia, which is a dimer consisting of the inducible Hif-1α subunit and the constitutively expressed Hif-1β subunit (Miraglia et al., 2016). Under aerobic conditions, Hif-1α remains inactive, while its activation is often associated with the function of Hif-1 transcription factor (Badowska-Kozakiewicz et al., 2016). Hif-1α is expressed in almost all cell types, including cancer cells, and it is recognized as a marker of poor survival in many solid tumors (Berg et al., 2016). In osteosarcoma, for example, Hif-1α accumulation help cancer cells adapt to and survive in hypoxic conditions, decreased overall survival and disease-free survival (Wei et al., 2016).

Hif-1α is an upstream gene involved in the transcriptional regulation of many genes, such as vascular endothelial growth factor (VEGF), transforming growth factor-β (TGF-β), stromal cell-derived factor-1 (SDF-1), basic fibroblast growth factor (bFGF), angiopoietin-1 (Ang-1), platelet-derived growth factor-BB (PDGF-BB), placental growth factor (PLGF) and stem cell factor (SCF; Zhang et al., 2016). Based on these, some studies have transfected the Hif-1α gene into BMSCs aiming to enhance the angiogenesis of BMSCs and subsequently produce satisfactory bone regeneration efficacy (Zou et al., 2011). Besides, evidence has been given that alternative expression of Hif-1α may regulate the migration of BMSCs (Choi et al., 2016). However, whether dysregulation of Hif-1α has a role in BMSCs transplantation have not been fully elucidated.

In the present study, Hif-1α was overexpressed in BMSCs by using lentivirus vectors aiming to explore the functions of Hif-1α on the survival of BMSCs under oxygen-glucose deprivation (OGD) condition. Further, the possible underlying mechanism through which modulated BMSCs survival was preliminarily found. In addition, whether the overexpression of Hif-1α in BMSCs has a gratifying outcome was detected in a rat model of middle cerebral artery occlusion (MCAO) *in vivo*.

## Materials and Methods

### BMSCs Isolation and Identification

BMSCs cells were isolated from Wistar rats (weighted 80–100 g; specific pathogen-free grade; Vital River Laboratories, Beijing, China) and cultured as previously described (Hussain et al., 2016). Rats were anesthetized by intraperitoneal (IP) injection of chloral hydrate (350 mg/kg), and the femur and tibia of rats were excised and bone marrow cells were slowly flushed out from the marrow cavity using DMEM medium (Gibco, Grand Island, NY, USA). Cell was cultured with DMEM supplemented with 10% fetal bovine serum (FBS, Hyclone, Logan, UT, USA). Non-adherent cells were removed after 48 h. Approximately 14 days later, the adherent stromal cells become confluent and were designated passage 0 (P0). The BMSCs were expanded and passaged to P3, at which point they were harvested and employed in the present study. Animal experiments performed in this study were all approved by the Animal Ethics Committee of Southern Medical University. Precautions were taken to minimize suffering and the number of animals used in each experiment.

BMSCs were identified by the expression of specific membrane markers using cytofluorimetric analysis with a flow cytometer (Beckman Coulter, CA, USA; Yu et al., 2013). Cells were incubated for 20 min at 4°C with the following antibodies: anti-CD29, anti-CD90, anti-CD105 and anti-CD45 (Biolegend, CA, USA).

### Hif-1α Transfection

For P1 BMSCs were infected by lentivirus with a multiplicity of infection (MOI) of eight (Liu et al., 2011). The BMSCs infected with Hif-1α recombinant lentivirus were defined as Hif-1α/GFP-BMSCs and the BMSCs infected with mock lentivirus were defined as GFP-BMSCs, which were used as a negative control (NC). To achieve the optimal gene transfer, polybrene (a final concentration of 8 μg/mL) was used. All BMSCs were expanded to three passes, and then used for transplantation. For Hif-1α silencing, Hif-1α siRNA (oligoID HSS104775: Invitrogen) were transfected into BMSCs using Lipofectamine 2000 (Invitrogen) according to the manufacturer’s instructions. Nonspecific siRNA (oligoID 12935-300: Invitrogen) was used as its NC. The efficiency of gene transduction was assessed with FACS and fluorescence microscope, qRT-PCR and Western blot.

### OGD Induction and Compound C Administration

BMSCs were incubated without glucose and exposed to hypoxic condition (5% CO_2_ and 95% N_2_). After 2 h of OGD induction, 24 h reperfusion with high-glucose DMEM in normoxia was carried out (Zhang J. et al., 2008; Yan et al., 2014).

Compound C, an inhibitor of AMPK, was purchased from Calbiochem (EMD Millipore, Billerica, MA, USA). BMSCs were cultured in completed medium in the presence or absence of Compound C (20 μM) for 2 h.

### Cell Viability Assay

Cell viability was assessed using a 3-(4,5-dimethylthiazol-2-yl)-2 5-diphenyl-2Htetrazolium bromide (MTT) colorimetric assay, according to standard methods described before (Wang et al., 2013). Briefly, cells were seeded into 96-well plants and cultured for 48 h. Twenty microliters of MTT solution (Sigma) with final concentration of 5 mg/mL was added into cells. After 4 h of incubation at 37°C, 150 μL dimethyl sulfoxide (DMSO, Sigma) was added and the absorbance was measured under a microplate reader (Bio-rod, Hercules, CA, USA) at 570 nm.

### Apoptosis Assay

Quantification of apoptosis was performed by using an Annexin V:FITC Apoptosis Detection Kit (BD Biosciences, San Jose, CA, USA), according to the manufacturer’s recommendations. Cells were resuspended in 200 μL Annexin-binding buffer containing 10 μL Annexin V-FITC and 5 μL PI. After 30 min of incubation in the dark, 300 μL phosphate buffer saline (PBS) were added into cells, and the apoptotic cells were distinguished under a flow cytometry (Beckman Coulter, CA, USA) immediately (Zhao et al., 2010).

Terminal deoxynucleotidyl (TUNEL) and 4′,6-diamidino-2-phenylindol (DAPI) staining were also performed to evaluate apoptosis. BMSCs were fixed with 4% paraformaldehyde, and then stained for TUNEL assay using the *in situ* Cell Death Detection Kit (Roche Diagnostics) according to the manufacturer’s protocol. To monitor the intact, condensed, and fragmented nuclei, the cells were counterstained with DAPI (Vector Laboratories, CA, USA; Bak et al., 2015). Images were acquired using a fluorescence microscope (Eclipse Ti, Nikon).

### qRT-PCR

Total RNA of cells were isolated by TRIzol reagent (Invitrogen, Carlsbad, CA, USA), cDNAs were synthesized from 0.1 mg of total RNAs using PrimeScript Reverse Transcriptase (Takara, Dalian, China). qRT-PCR was performed using a SYBR Green PCR Mix (Qiagen) on QuantStudio 6 Flex Realtime PCR system (Applied Biosystems, Carlsbad, CA, USA; Zhang et al., 2013). Data were normalized to the β-actin expression and calculated by the 2^−ΔΔCt^ method.

### Western Blot

BMSCs were lysed in Radio Immunoprecipitation Assay (RIPI, Beyotime, Shanghai, China) for protein extraction, and the protein concentration was measured by the Bradford assay (Thermo, Hercules, CA, USA). Protein samples were separated by sodium dodecyl sulfate-polyacrylamide gel electrophoresis (SDS-PAGE) and transferred onto nitrocellulose membranes. Membranes were then incubated with 5% non-fat dried milk for 1 h at room temperature, and incubated with primary antibodies against p-AMPK, AMPK, cleaved caspase-9, caspase-9, Bcl-2, Bcl-xl, Bax, LC3 I, LC3 II, p-mTOR, mTOR, Beclin1, p62, Hif-1α (1:1000; all from cell signaling technology), GAPDH (1:1000; Chemicon International) and β-actin (1:1000; Santa Cruz), overnight at 4°C. After washed in TBST buffer, the membranes were incubated with the appropriate secondary antibodies (1:5000; Odyssey, LI-COR) for 2 h. Positive signals were acquired with the Odyssey infrared imaging system and analyzed as specified in the Odyssey software manual (Yan et al., 2014). GAPDH and β-actin were used as internal references.

For *in vivo* detection, 100 g left penumbra was removed from rats and placed in the grinder. Following by adding 5 mL RIPI (Beyotime), the penumbra was ground to obtain protein samples. After electrophoresis and transmembrane, membranes were probed by primary antibodies: TNF-α, IL1β, IL-6, BDNF, VEGF (1:1000; all from cell signaling technology) and GAPDH (1:1000; Chemicon International) overnight. The appropriate secondary antibodies (1:5000; Odyssey, LI-COR) were then added and incubated for 2 h. Protein bands were acquired and assayed as above mentioned.

### Ultrastructural Analysis by Transmission Electron Microscopy

Cells were fixed in 4% paraformaldehyde and 2% glutaraldehyde in 0.1 M PBS at pH 7.4 overnight at 4°C. After osmium tetroxide postfixation and alcohol dehydration, the samples were embedded in epoxy resin (Araldite, Fluka, Switzerland) and ultrathin sections (60 nm) were obtained using an ultramicrotome (Ultracut, Reichert, Vienna, Austria). The samples were then stained with uranyl acetate and lead citrate, and viewed in the transmission electron microscope (TEM 1230EX, JEOL, Japan) at 60 kV (Kathuria et al., 2014).

### MCAO

Wistar rats, belonging to the same strain with those used for collecting BMSC, were anesthetized by IP injection of choral hydrate (350 mg/kg). Body temperature was maintained at 37 ± 0.5°C using a heating pad (RWD Life Science, Shenzhen, China). An 4-0 suture (Covidien, Mansfield, MA, USA) with round tip and silicon coating was inserted from the left external carotid artery into the middle cerebral artery in rats (Yang and Betz, 1994). The success of the surgery was verified by detection the surface cerebral blood flow using a Laser Doppler flowmetry (Moor LAB, Moor Instruments, Devon, UK). After 2 h of suture insertion, the rats were anesthetized again and the suture was withdrawn to perform a reperfusion. The rats in the control group underwent a sham operation without suture insertion.

### BMSCs Transplantation

The BMSCs transplantation was performed 24 h after transient MCAO. BMSCs (1 × 10^6^ in 3 μL DMEM) were stereotactically injected into the striatum of the ipsilateral hemisphere. To maximize engraftment of all injected BMSCs, the needle was not disconnected from the striatum of the ipsilateral hemisphere for 5 min after the injection. The same amount of DMEM without cells was injected as the control.

### Survival of Transplanted BMSCs

The survival rates of transplanted BMSCs after MCAO surgery were assessed at 14 days post-injury. Midsagittal sections prepared from the injured portion of the ipsilateral hemisphere striatum. The numbers of GFP-positive BMSCs in these sections were counted as described in the section on semi-quantitative analysis of tissue staining. The cells were observed under a fluorescence microscope (Eclipse Ti, Nikon) with the appropriate excitation wavelengths (Liu et al., 2011).

### Measurement of Cerebral Infarction Volume

Cerebral infarction volume in rats was evaluated after 14 days of BMSCs transplantation. The fresh brain slices were incubated in a 2% solution of 2, 3, 5-triphenyltetrazolium chloride (TTC, Sigma, St. Louis, MO, USA) in PBS at room temperature for 20 min. The color of the ischemic area was white and of non-ischemic area was red. Crossly visible infarction zones in the slices were measured by image analysis software (National Institutes of Health, Bethesda, MD, USA) and final infarction volume was calculated as described previously (Frieler et al., 2011).

### Specific Behavioral Tasks

Neurological function was assessed at the beginning and 14 days after BMSCs transplantation, using a modified Neurological Severity Score (mNSS; Liu et al., 2011). The mNSS is a composite of the motor (muscle status and abnormal movement), sensory (visual, tactile, and proprioceptive) and reflex tests. The neurological function was graded on a scale of 0–18 (normal score 0, maximal deficit score 18).

Morris swim tasks (Crawley, 1999) have been standardized to measure spatial navigation learning and memory in rats. In brief, 14 days after BMSCs transplantation, each rats learned to swim in a circular pool of water to locate a submerged hidden platform. The circular pool (150 cm in diameter) was filled with 30 cm deep of water (24–26°C). Black food coloring was added in order to avoid rats to see the platform and the floor of pool. A circular transparent Plexiglas platform (10 cm in diameter) was submerged 1.5 cm below the surface of the water in the center of an arbitrarily defined quadrant of the pool (southwest) and remained in the same position throughout the testing. At the first experimental day, rats were allowed to swim in the pool in the absence of the platform for 60 s. During the next five consecutive days, four trails per day with the platform in place were used for rats. The time interval between each trial sessions was 30 min. The swim path lengths were recorded. On the sixth day, the probe test was carried out by removing the platform and allowing each rat to swim freely for 60 s. The time required for the rat spent swimming in the target quadrant (where the platform was located during hidden platform training) as well as the times crossing over the platform site of rats were measured and recorded respectively. The mean data from daily training trial tests and data from the tests at the last day were used for statistical analysis.

### Statistical Analysis

All experiments were repeated more than three times. The results were presented as the mean ± standard deviation (SD). Statistical analyses were performed using GraphPad Prism 5.0 software (GraphPad Software, San Diego, CA, USA) by a one-way analysis of variance (ANOVA) or two-way repeated-measures ANOVA. A *P*-value of < 0.05 was considered statistically significant.

## Results

### BMSCs Identification and Efficiency of Gene Transduction

**Figure 1:**
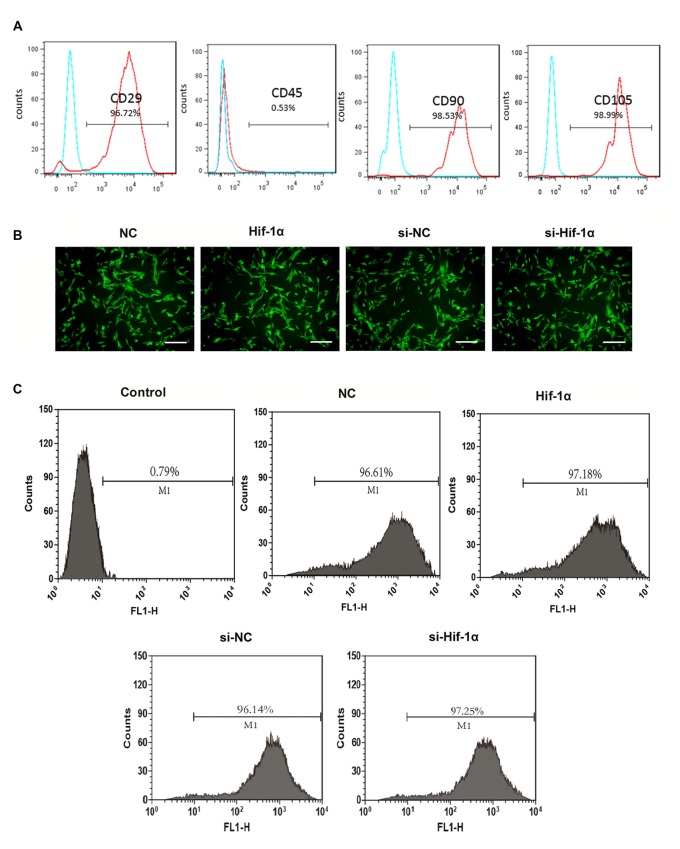
**Bone mesenchymal stem cells (BMSCs) identification and efficiency of transduction. (A)** Flow cytometry analysis for CD29, CD45, CD90 and CD105. **(B)** The immunofluorescent micrograph of BMSCs infected with hypoxia-inducible factor 1α (Hif-1α)-GFP lentiviral vector, Hif-1α siRNA or their negative controls (NCs). Scale bar = 200 μm. **(C)** Efficiency of gene transduction was detected by flow cytometry analysis.

**Figure 2:**
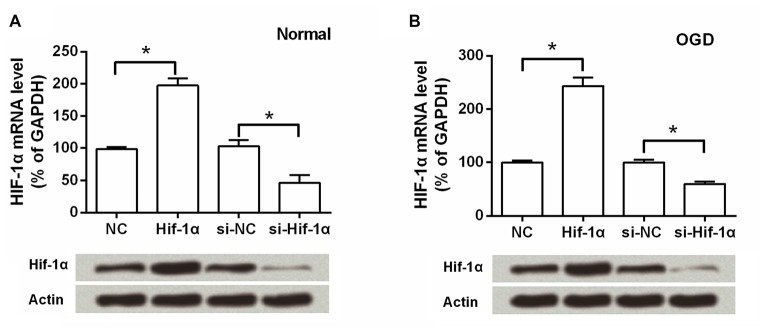
**Hif-1α expression was altered post-transfection with or without exposure to oxygen-glucose deprivation (OGD) conditions.** Both the mRNA and protein levels of Hif-1α were measured by qRT-PCR and Western blot post-transfection under **(A)** normal and **(B)** OGD conditions. *n* = 3. *P < 0.05.

As shown in Figure 1A, the BMSCs were 96.72% pure for CD29, 98.53% for CD90 and 98.99% for CD105. The contaminated population of BMSCs positive to CD45 was 0.53%. BMSCs were then transfection with Hif-1α-GFP vector, Hif-1α siRNA or their corresponding controls. The efficiency of gene transduction with Hif-1α-GFP vector was similar to that of NC (97.18% vs. 96.61%), and Hif-1α siRNA was similar to that of siNC (97.25% vs. 96.14%; Figures 1B,C). Further, we detected the expression of Hif-1α under normal and OGD conditions post-transfection. We found that both the mRNA and protein levels of Hif-1α were increased in BMSCs which were infected with Hif-1α-GFP vector when compared with those in BMSCs infected with NC (*P* < 0.05; Figures 2A,B). Meanwhile, Hif-1α expression was decreased in Hif-1α-silencing BMSCs when compared with those in siNC group (*P* < 0.05). These data indicated that Hif-1α expression was successfully altered with or without exposure to OGD conditions.

### Hif-1α Overexpression Promoted BMSCs Viability and Suppressed Apoptosis

**Figure 3:**
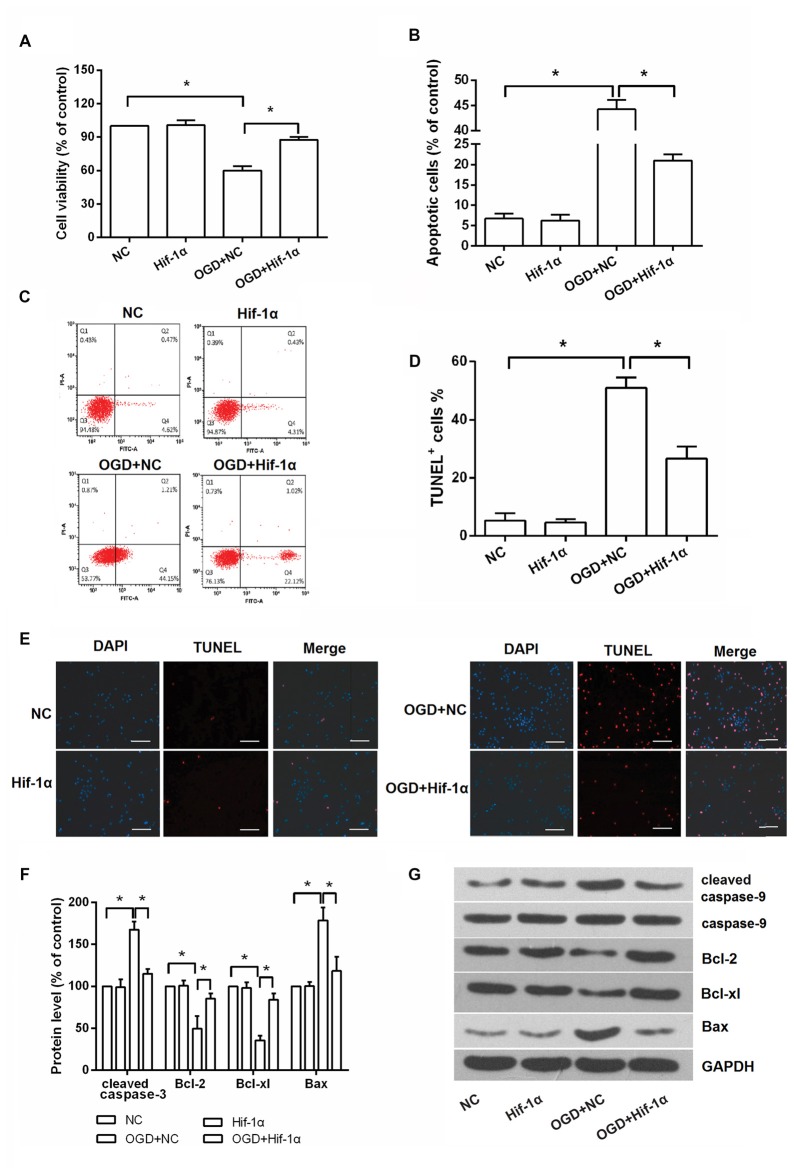
**Hif-1α overexpression protected BMSCs from OGD induced injury.** BMSCs were transfected with Hif-1α-GFP lentiviral vector or a mock lentivirus (NC), and then exposed to OGD condition. **(A)** Cell viability, **(B–E)** apoptosis and **(F,G)** apoptosis associated factors expression were respectively detected by 3-(4,5-dimethylthiazol-2-yl)-2 5-diphenyl-2Htetrazolium bromide (MTT), flow cytometry, terminal deoxynucleotidyl (TUNEL) staining and Western blot. Scale bar = 100 μm. *n* = 3. *P < 0.05.

To assess the effects of Hif-1α overexpression on BMSCs viability and apoptosis *in vitro*, an OGD model was applied. BMSCs viability was remarkably reduced after exposure in OGD condition, whereas Hif-1α overexpression could alleviate this reduction (*P* < 0.05; Figure 3A). In contrast, cellular apoptosis was significantly increased by OGD; surprisingly, this increase was also alleviated by Hif-1α overexpression (*P* < 0.05; Figures 3B–E). Next, the protective impacts of Hif-1α on BMSCs at the molecular level were verified. Up-regulations of cleaved caspase-9, Bax and down-regulations of Bcl-2, Bcl-xl were found in BMSCs after OGD induction (*P* < 0.05). These dysregulations were all partly abolished by Hif-1α overexpression (*P* < 0.05; Figures 3F,G). Thus, Hif-1α might protect BMSCs from OGD induced injury.

### The Involvement of AMPK and mTOR in OGD Induced Injury

**Figure 4:**
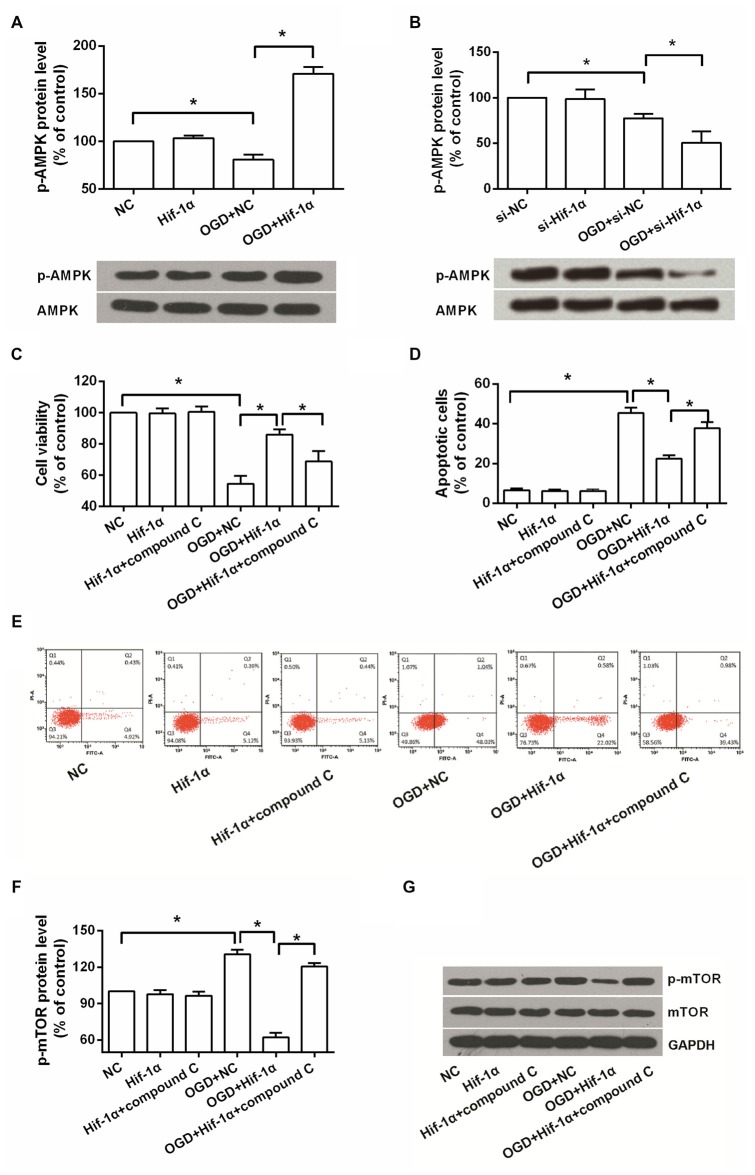
**Hif-1α overexpression protected BMSCs from OGD induced injury via regulating AMPK and mTOR.** BMSCs were transfected with Hif-1α-GFP lentiviral vector, Hif-1α siRNA or their NCs, and then exposed to OGD condition followed by compound C administrate. **(A,B)** Phosphorylation of AMPK was detected by Western blot. **(C–E)** Cell viability and apoptosis were measured by MTT and flow cytometry. **(F,G)** Phosphorylation of mTOR was determined by Western blot. *n* = 3. *P < 0.05.

Western blot analysis revealed that the level of p-AMPK in BMSCs was decreased after OGD induction, while was notably increased when Hif-1α was overexpressed (*P* < 0.05) and was decreased when Hif-1α was knocked down (*P* < 0.05; Figures 4A,B). An AMPK inhibitor, compound C, was used to confirm whether AMPK was implicated in the functions of Hif-1α on BMSCs. We found that, adding of compound C could partly recover the alleviative impacts of Hif-1α overexpression on OGD induced injury (*P* < 0.05; Figures 4C–E). mTOR is an important downstream target of AMPK, its protein expression was also detected by Western blot. OGD increased p-mTOR level and Hif-1α overexpression abolished this increase; more importantly, addition of compound C re-increased p-mTOR protein expression (*P* < 0.05; Figures 4F,G). These data indicated Hif-1α protected BMSCs from OGD induced injury via modulation of AMPK and mTOR.

### Hif-1α Overexpression Enhanced Autophagy

**Figure 5:**
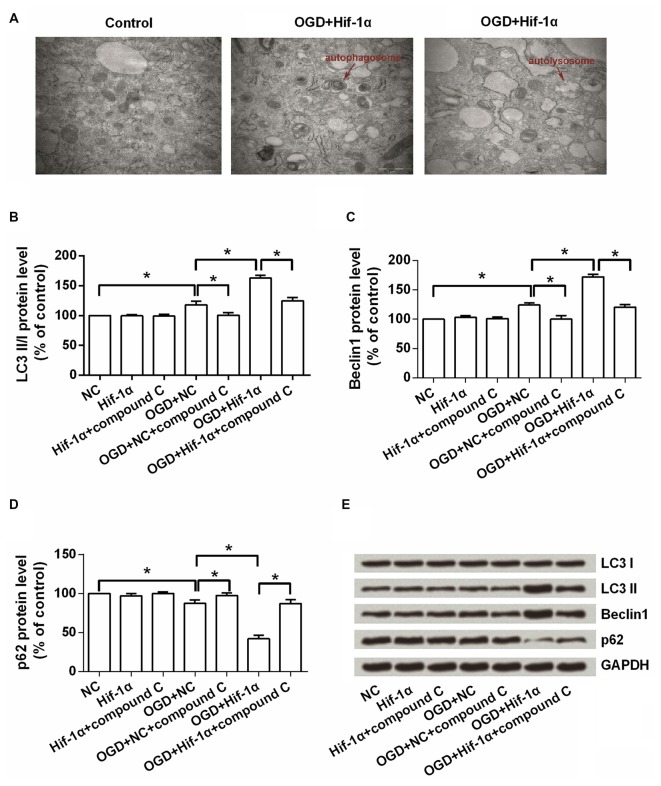
**Hif-1α overexpression promoted autophagy.** BMSCs were transfected with Hif-1α-GFP lentiviral vector or a mock lentivirus (NC), and then exposed to OGD condition followed by compound C administrate. **(A)** The autophagosome and autolysosome were observed by transmission electron microscopy. Scale bar = 1 μm. **(B–E)** The expression levels of LC3 I, LC3 II, Beclin1 and p62 were assessed by Western blot. *n* = 3. *P < 0.05.

We further explored the effects of Hif-1α overexpression on autophagy in BMSCs. Electron microscopy images of BMSCs revealed that more autophagosome and autolysosome were observed in Hif-1α overexpressed BMSCs after OGD induction (Figure 5A). Western blot analysis showed that (Figures 5B–E), the levels of LC3 II and Beclin1, two autophagy markers, were significantly increased by OGD (*P* < 0.05). Hif-1α overexpression enhanced the increase of these two proteins, whereas compound C abolished this enhancement (*P* < 0.05). Conversely, p62 was down-regulated by OGD, and this down-regulation was enhanced by Hif-1α overexpression, while was abolished by compound C (*P* < 0.05). p62 is conjugated to LC3 and thus serving as an alternative indicator of autophagy (Bjørkøy et al., 2005). Overall, these results indicated a promoting role of Hif-1α in BMSCs autophagy.

### Hif-1α Overexpression Reduced Brain Infarct Volume of MCAO Rats

**Figure 6:**
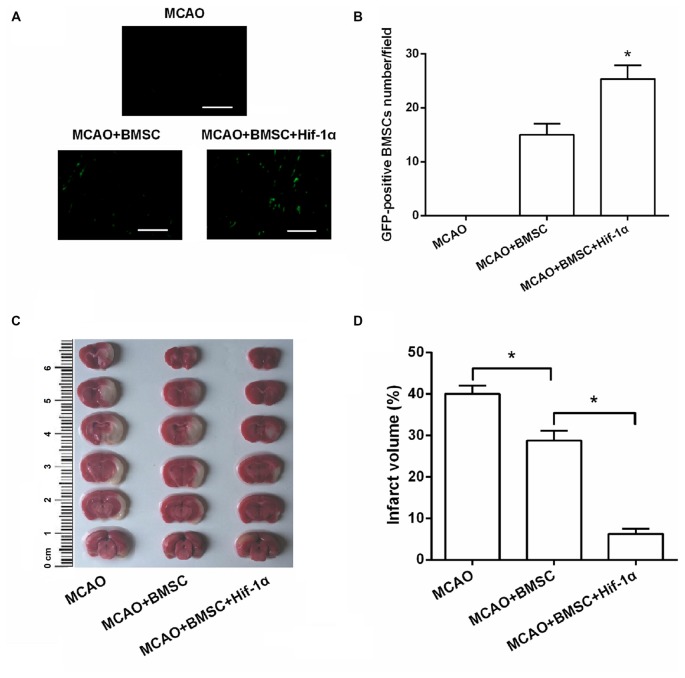
**Hif-1α overexpression reduced brain infarct volume of middle cerebral artery occlusion (MCAO) rats.** The modified BMSCs transplanted into the brain of MCAO model of rats. **(A,B)** GFP-positive BMSCs number was counted under a fluorescence microscope. Scale bar = 200 μm. **(C,D)** Cerebral infarction volume in rats was evaluated. *n* = 5. *P < 0.05.

In Figures 6A,B, GFP-positive BMSCs were not detected in the MCAO group, whereas were observed after BMSCs transplantation. Of note, GFP-positive BMSCs number was greatly increased after transplantation with Hif-1α overexpressed BMSCs (*P* < 0.05), demonstrating the survival of the transplanted BMSCs was modified by Hif-1α overexpression. Brain infarct volume was detected at 14 days post-transplantation. Results showed that BMSCs significantly reduced brain infarct volume, and Hif-1α overexpression strengthened this reduction (*P* < 0.05; Figures 6C,D).

### Hif-1α Overexpression Improved Neurobehavioral Outcome of MCAO Rats

**Figure 7:**
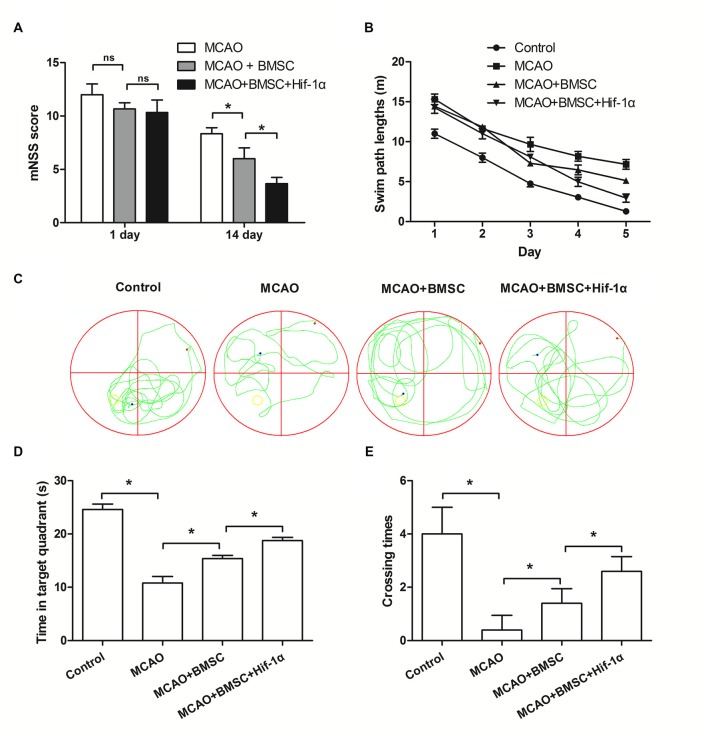
**Hif-1α overexpression improved neurobehavioral outcome of MCAO rats.** The Hif-1α-modified BMSCs transplanted into the brain of MCAO model of rats. **(A)** Neurological function was assessed using a modified Neurological Severity Score (mNSS). **(B)** Swim path lengths. **(C)** Exploration patterns. **(D)** Times for the rats spend in the target quadrant. **(E)** Times for crossing over the platform were assessed by Morris swim tasks. *n* = 5. *P < 0.05.

Next, mNSS and Morris swim tasks were performed to assess the motor, sensory and memory functions of MCAO rats. Neurological score showed that neurobehavioral outcomes were greatly improved in the BMSCs transplantation group, and Hif-1α overexpression further enhanced this improvement (*P* < 0.05; Figure 7A). Morris swim tasks results given in Figure 7B showed that, path lengths were longer in MCAO rats than Control rats (*F* = 11.122; *P* < 0.05), but shorter in MCAO + BMSC rats than MCAO rats (*F* = 5.336; *P* < 0.05). MCAO rats transplanted with Hif-1α-modified BMSC had shorter path lengths than MCAO + BMSC rats (*F* = 4.231; *P* < 0.05). In addition, the time required for rats spent in the target quadrant and crossing over the platform was less in MCAO rats than Control rats (*P* < 0.05), and was more in MCAO + BMSC rats than MCAO rats (*P* < 0.05; Figures 7C–E). MCAO rats transplanted with Hif-1α-modified BMSC spend more times in the target quadrant and crossing over the platform (*P* < 0.05). Together, these data provided *in vivo* evidences that Hif-1α overexpression improve neurobehavioral outcome of MCAO rats.

### Hif-1α Overexpression Inhibited Pro-Inflammatory Cytokines Generation While Promoted Neurotrophin Secretion

**Figure 8:**
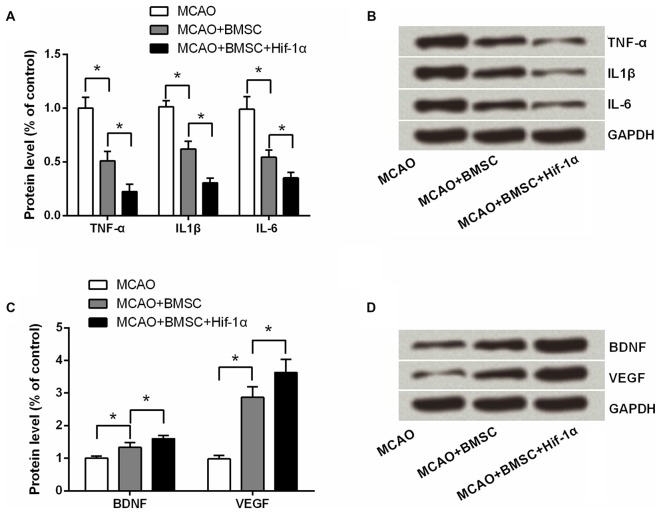
**Hif-1α overexpression inhibited pro-inflammatory cytokines generation while promoted neurotrophin secretion.** The modified BMSCs transplanted into the brain of MCAO model of rats. Left penumbra was removed from rats, and tissue protein samples were used for detecting **(A,B)** TNF-α, IL1β and IL-6 levels, and **(C,D)** BDNF and vascular endothelial growth factor (VEGF) levels by Western blotting. *n* = 3. *P < 0.05.

Further, to ask whether the Hif-1α overexpression could alter the expression of pro-inflammatory cytokines and neurotrophin, we tested TNF-α, IL1β, IL-6, BDNF and VEGF expressions by using Western blotting analysis. As results showed in Figures 8A,B, all these pro-inflammatory cytokines were down-regulated after MCAO rats were transplanted with 1 × 10^6^ BMSCs (*P* < 0.05), and the Hif-1α-engineered BMSCs exhibited a stronger effects than the original BMSCs on these factors down-regulation (*P* < 0.05). Results in Figures 8C,D showed that both BDNF and VEGF were up-regulated by BMSCs transplantation; as expected, Hif-1α-engineered BMSCs could further up-regulated these two expressions (*P* < 0.05). These data suggested Hif-1α overexpression improve neurobehavioral outcome of MCAO rats possibly via inhibition of inflammatory response and promotion of neurotrophin secretion.

## Discussion

Our present study suggested that overexpression of Hif-1α promoted BMSCs viability and suppressed apoptosis after OGD induction *in vitro*. Mechanistic study demonstrated that, these protective functions of Hif-1α overexpression on OGD induced injury in BMSCs might be via modulation of AMPK and mTOR, and via promoting autophagy. Transplantation of the overexpressed Hif-1α of BMSCs into the MCAO rat significantly reduced brain infarct volume and improved neurobehavioral outcome. In addition, Hif-1-overexpressing BMSCs remarkably inhibited pro-inflammatory cytokines generation while promoted neurotrophin secretion. These results provided preliminary evidences that Hif-1α engineering in BMSCs might be a therapeutic strategy for cerebral infarction by improving its efficacy.

Donor BMSCs death after transplantation is one of the serious obstacles impacting on the outcome of cell therapy for cerebral infarction. Modification of BMSCs with several genes has been found to enhance the survival of the implanted BMSCs, such as Bcl-2 (Li et al., 2007), HO-1 (Tang et al., 2005) and AKT (Mangi et al., 2003). In the current study we found that overexpression of Hif-1α also could improve BMSCs survival by controlling of cellular viability and apoptosis. In fact, previous studies have determined that Hif-1α was up-regulated under hypoxic conditions, and that affected cellular survival (Eliasson and Jönsson, 2010). Lampert et al. (2016) demonstrated that overexpression of Hif-1α could significantly increase BMSCs proliferation while reduce the apoptotic rate. All these above suggests that overexpression of Hif-1α supports BMSCs survival. In the present study, we also found that Hif-1α overexpressed BMSCs reduced the infract volume and improved neurobehavioral outcome of MCAO rats. The infarcted volumes increased along with duration of ischemia (Zhang et al., 2013), which suggested that permanent ischemia is an ongoing process that can be decelerated by Hif-1α overexpression. In addition, in the present study, BMSCs remarkably inhibited pro-inflammatory cytokines generation and promoted neurotrophin secretion, these data further confirmed the protective roles of Hif-1α-overexpressing BMSCs in rat MCAO stroke model.

AMPK is an upstream of mTOR which is a metabolic sensor and activated by low energy. AMPK is an important receptor that helps cells recognize the changes in energy regulation, while mTOR functions as a vital signaling complex in many cellular processes, such as cell proliferation, cell cycle, apoptosis and autophagy (Kim et al., 2011). Interestingly, AMPK activity was shown to be important for Hif-1α transcriptional activity under hypoxic and normoxic conditions (Woo et al., 2015). In the current study, under OGD condition Hif-1α overexpression increased the phosphorylation of AMPK while suppressed mTOR activation. Compound C, an AMPK inhibitor, could partly abolish the improving functions of Hif-1α overexpression on OGD induced injury in BMSCs. It is plausible that Hif-1α overexpression protects BMSCs from OGD via activation of AMPK and inactivation of mTOR.

Autophagy can be induced by multiple signaling pathways that related with cell growth, proliferation, survival and death (Wu et al., 2016). Autophagy is essential for scavenging damaged organs in cells and has been described as a Hif-1α-dependent adaptive response (Zhang H. et al., 2008). Knock-down of Hif-1α resulted in significantly decreased level of autophagy (Lin et al., 2016). Consistent with those findings, results in this study marked a promoting role of Hif-1α in BMSCs autophagy, as more autophagosome and autolysosome, as well as dysregulations of autophagy markers were observed in Hif-1α-overexpressing BMSCs. Besides, it seemed that the autophagy induced by Hif-1α overexpression was abolished by inhibition of AMPK. Actually, mTOR and AMPK have been identified as major regulators of autophagy. Activated AMPK negatively regulates mTOR and thereby enhances autophagy flux. AMPK/mTOR signaling in autophagy has become the hot spot in recent years (Kim et al., 2011). However, the hypothesis of Hif-1α modulates autophagy by AMPK/mTOR signaling still need enormous amount of experimental studies.

In conclusion, our observation indicated that Hif-1α might be contributed in the survival of BMSCs by regulating the activation of AMPK and mTOR, as well as by promoting autophagy. These findings might provide novel evidence supporting modification of BMSCs has benefits on treatment for cerebral infarction.

## Author Contributions

BL, FL and XJ designed the experiments; BL, JH, JF, LX and CS performed the experiments; TH, ZZ and ZF performed data collection and analysis; BL, FL, JH, JF, LX, CS, TH, ZZ and ZF wrote the manuscript together; XJ revised the manuscript and provided financial support. All of them performed the final approval of the manuscript.

## Conflict of Interest Statement

The authors declare that the research was conducted in the absence of any commercial or financial relationships that could be construed as a potential conflict of interest.

## Abbreviations

Ang-1: angiopoietin-1
bFGF: basic fibroblast growth factor
BMSCs: bone mesenchymal stem cells
DAPI: 4^′^,6-diamidino-2-phenylindol
DMSO: dimethyl sulfoxide
Hif-1: hypoxia-inducible factor 1
MCAO: middle cerebral artery occlusion
mNSS: modified Neurological Severity Score
MOI: multiplicity of infection
MTT: 3-(4,5-dimethylthiazol-2-yl)-2 5-diphenyl-2Htetrazolium bromide
NC: negative control
OGD: oxygen-glucose deprivation
PBS: phosphate buffer saline
PDGF-BB: platelet-derived growth factor-BB
PLGF: placental growth factor
SCF: stem cell factor
SD: standard deviation
SDF-1: stromal cell-derived factor-1
SDS-PAGE: sodium dodecyl sulfate-polyacrylamide gel electrophoresis
TGF-β: transforming growth factor-β
TTC: triphenyltetrazolium chloride
TUNEL: terminal deoxynucleotidyl
VEGF: vascular endothelial growth factor.
